# *CIAS1-*associated autoinflammatory syndrome first diagnosed at age 48 years: a case report

**DOI:** 10.1186/1546-0096-13-S1-P211

**Published:** 2015-09-28

**Authors:** M Fasshauer, S Borte, A Hauenherm, E Braun, H Reichenbach, M Borte

**Affiliations:** 1 Klinik für Kinder- und Jugendmedizin, Klinikum St. Georg gGmbH Leipzig, Akademisches Lehrkrankenhaus der Universität Leipzig, Germany; 2 ImmunDefektCentrum Leipzig (IDCL) am Klinikum St. Georg gGmbH Leipzig, Germany; 3 Mitteldeutscher Praxisverbund Humangenetik, Leipzig, Germany

## 

Cryopyrin-associated periodic syndromes are a group of autoinflammatory syndromes caused by mutations of the *CIAS1*gene (currently named *NLRP3*), and are characterized by periodic attacks of an urticaria-like rash, fever, headache, conjunctivitis and arthralgia. Often patients present in early childhood, but the great diversity of manifestations and the difficulties in genetic analyses make the diagnosis of these diseases a challenge. The authors describe the clinical features of a male patient who presented first symptoms approximately at age 23 years. with recurrent fevers up to 40°C, urticaria-like rash, orbital swelling, headache and fatigue. *CIAS1*-associated autoinflammatory syndrome was diagnosed at age 48 years. and confirmed genetically (mutation: c.598G>A (p.Val200Met) in Exon 3 of *NLRP3* (*CIAS1*)-gene.

Written informated consent for publication of their clinical details was obtained from the patient/parent/guardian/relative of the patient.

